# How Safe Are the Laparoscopic and Robotic Graspers? Evaluation of the Novel Avatera Robotic Surgical System: An Acute In Vivo Study on a Porcine Model

**DOI:** 10.5152/tud.2023.23127

**Published:** 2023-11-01

**Authors:** Vasileios Tatanis, Anastasios Natsos, Arman Tsaturyan, Athanasios Vagionis, Angelis Peteinaris, Solon Faitatziadis, Kristiana Gkeka, Konstantinos Pagonis, Mohammed Obaidat, Eirini Anaplioti, Dimitra Koumoundourou, Vassiliki Bravou, Theofanis Vrettos, Panagiotis Kallidonis, Evangelos Liatsikos

**Affiliations:** 1 Department of Urology, University of Patras, Patras, Greece; 2 Department of Urology, Erebouni Medical Center, Yerevan, Armenia; 3 Department of Pathology, University Hospital of Patras, Patras, Greece; 4 Department of Anesthesiology and ICU, University of Patras, Patras, Greece; 5 Department of Urology, Medical University of Vienna, Vienna, Austria

**Keywords:** Avatera robotic system, bowel, injury, robotic graspers, robotic-assisted laparoscopic surgery

## Abstract

**Objective:**

To evaluate the tissue injury caused by the force applied by the robotic-assisted graspers of avatera robotic surgical system on bowel tissue.

**Methods:**

An experimental in vivo porcine model with 1 pig was conducted. After a standard transperitoneal setup of the avatera robotic surgical system, different laparoscopic and robotic graspers were used on the bowel with maximum force applied each time. Robotic atraumatic grasper, laparoscopic right angle grasper, laparoscopic curved grasper, and laparoscopic atraumatic grasper were used. After using all graspers, the pig was sacrificed. The bowel segments were resected and sent for histological analysis.

**Results:**

The pathologist reported that all the graspers caused signs of acute inflammation without any irreversible damage or signs compatible with ischemia of the tissue. No significant difference in histology was observed between the graspers.

**Conclusion:**

No permanent damage was caused by graspers, except for acute, reversible inflammation. Concluding, the avatera grasper could be safe to use on bowel segments, independent of the applied pressure.

**Main Points**

- One robotic atraumatic grasper and 3 laparoscopic graspers were applied for 5 minutes on a bowel segment with maximum force.
- All grasper-applied points were similar without significant features suggestive of ischemic necrosis.
- The avatera system is a novel promising surgical tool in robotic-assisted surgeries.

## Introduction

During the last decades, minimally invasive surgery has been marked by a huge revolution. The laparoscopy was the first representative expelling open surgery. Afterward, robotic-assisted laparoscopic surgery (RALS) or robotic minimally invasive surgery (RMIS) became the gold standard for most oncologic urologic surgeries. The ability to mobilize, hold, or dissect tissue during the surgery is achieved with graspers, making them necessary for the performance of laparoscopic and RALS. While no surgery is feasible without them, one disadvantage of most laparoscopic graspers is the danger of injuring delicate gastrointestinal tissues and causing irreversible damage at the cellular level. There is a variety of grasper geometries and teeth profiles, with or without fenestration and more. All these differences in grasper characteristics may also lead to varying degrees of tissue damage.

Inappropriate use of laparoscopic graspers can lead to serious iatrogenic complications including bowel perforation, serosal damage, and postoperative adhesion formation.^1^ An overall incidence of 0.8% non-access-related bowel injuries has been described. More precisely, serosal injury of the intestine or stomach was identified in 0.6% of patients, while bowel perforation was identified in about 0.2% of cases.^1^ Despite the low incidence, bowel perforation is associated with a high morbidity and mortality rate (as high as 3.6%).^2^ Bowel perforation, postoperative adhesion formation, and serosal tears are often due to improper use of laparoscopic graspers, while many mechanical injuries usually occur outside the laparoscopic visual field. Pulling and pressing the bowel tissues outside of the surgical view may lead to inadvertent injuries.

Although manufacturers market “atraumatic” forceps to ensure the safety of manipulating delicate bowel tissue, the damage caused is not in all cases well investigated.^3^ A major drawback especially in RMIS is the loss of haptic evaluation of the handled tissue. Contrary to the traditional open surgery, where the surgeon controls his force applied on the tissue, in RIMS, the surgeon must have in-depth knowledge of the forces applied through the machine on the tissue.^4^

Until now, most studies investigating bowel injury used laparoscopic graspers or graspers by the da Vinci surgical system. In this in vivo model, we aimed to investigate the possibility of injury using laparoscopic graspers and grasper of the avatera robotic system, a new robotic system that has been recently launched by avatera medical  GmbH (Jena, Germany).

## Material and Methods

**Figure 1.**
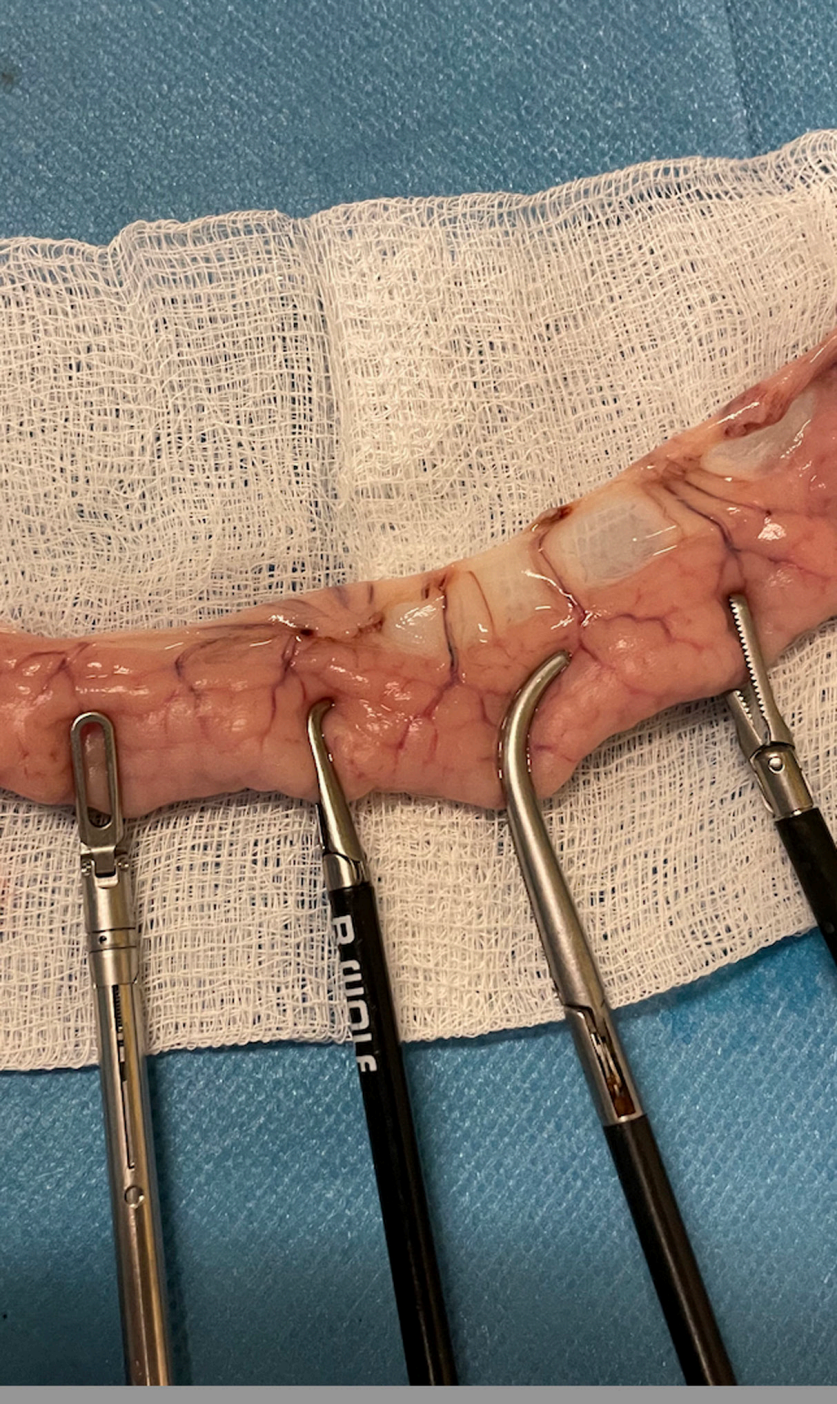
The application of the graspers on the bowel segment (replicated ex vivo model).

An experimental in vivo porcine model was utilized. The experiment was performed at the Urology Department of University of Patras. Ethical approval of the in vivo experimental and clinical studies was obtained before the initiation of the experiment (Approval no. ΠΔΕ/ΔΚ/279940/1239) from University of Patras. One female pig weighing more than 30 kg was included. The initiation of anesthesia was achieved by injecting ketamine (5 mg/kg), xylazine (1 mg/kg), and atropine sulfate. Intravenous injection of propofol 5% was utilized for the maintenance of anesthesia throughout the whole procedure. Trocars were placed using the standard protocol for intraperitoneal access. The robotic system was docked. A bowel segment was selected and mobilized. After the mobilization, different graspers were applied with maximum force on the bowel segment at a 5 cm distance from each other. The used graspers were robotic atraumatic grasper (avateramedical GmbH, Jena, Germany), laparoscopic right angle grasper (Richard Wolf GmbH, Knittlingen, Germany), laparoscopic curved grasper (Richard Wolf GmbH), and laparoscopic atraumatic grasper (Richard Wolf GmbH) (Figure 1). The application time was 5 minutes under the maximal force of each grasper. After testing all graspers, rapid laparotomy was performed. The intestinal area of interest was resected and immediately placed into formalin. Each grasper application point was marked using a different type of stitch on the free-of-grasper interval segments. In the end, the pig was sacrificed. The resected bowel segment was sent for histological examination.

### Avatera System

The avatera system is a newly introduced robotic surgical system consisting of 2 separate subunits: the control and robotic units. The former has many components including a flexible seat for the surgeon and the controllers to handle the robotic arms. The slender eyepiece constitutes an innovation of this subunit that enables improved and unobstructed communication between the surgeon and the other members of the surgical team. The robotic unit consists of 4 arms to apply the robotic instruments. The endoscope may be connected to the second or third arm, depending on the operation performed. The available instruments, including Metzenbaum scissors, needle holder, Maryland dissector, and atraumatic grasper, can be applied to the remaining 3 arms of the robotic system. Moreover, the use of bipolar-only energy is another particular characteristic of the avatera system. It is available to be applied to Maryland dissector and Metzenbaum scissors for coagulation and cutting, respectively.^5^

## Results

**Figure 2.**
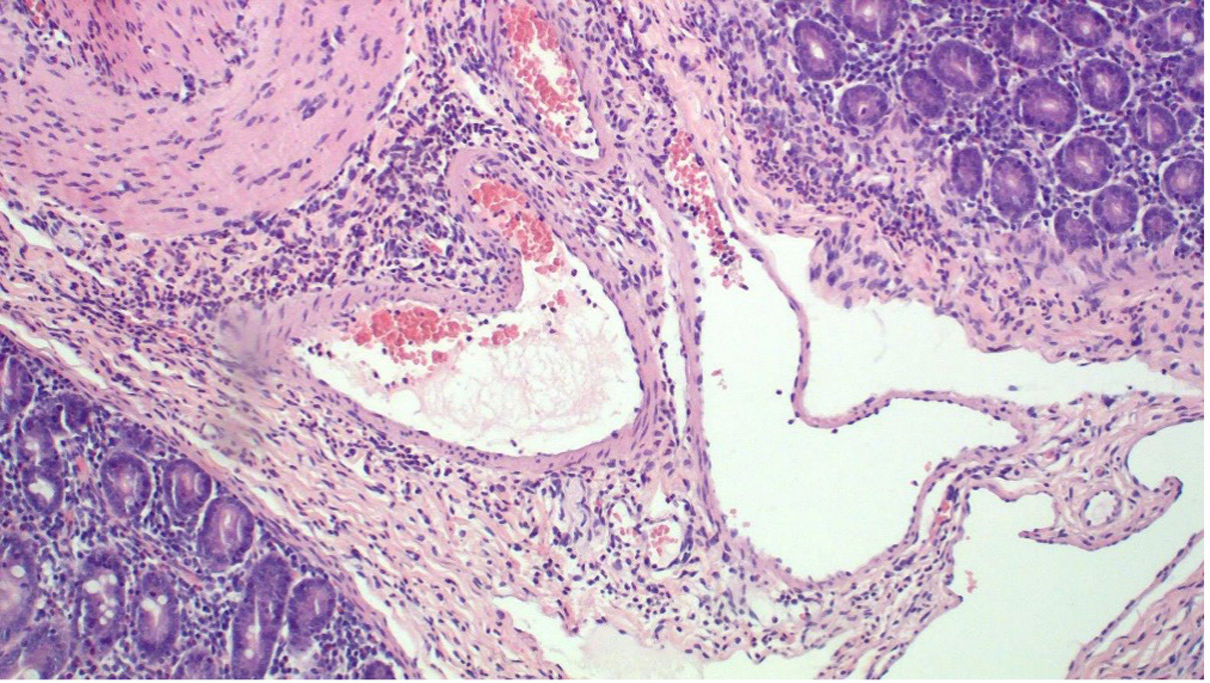
Whole-mount specimen of the small intestine showing mucosal inflammation as well as vascular dilatation in the submucosa.

**Figure 3.**
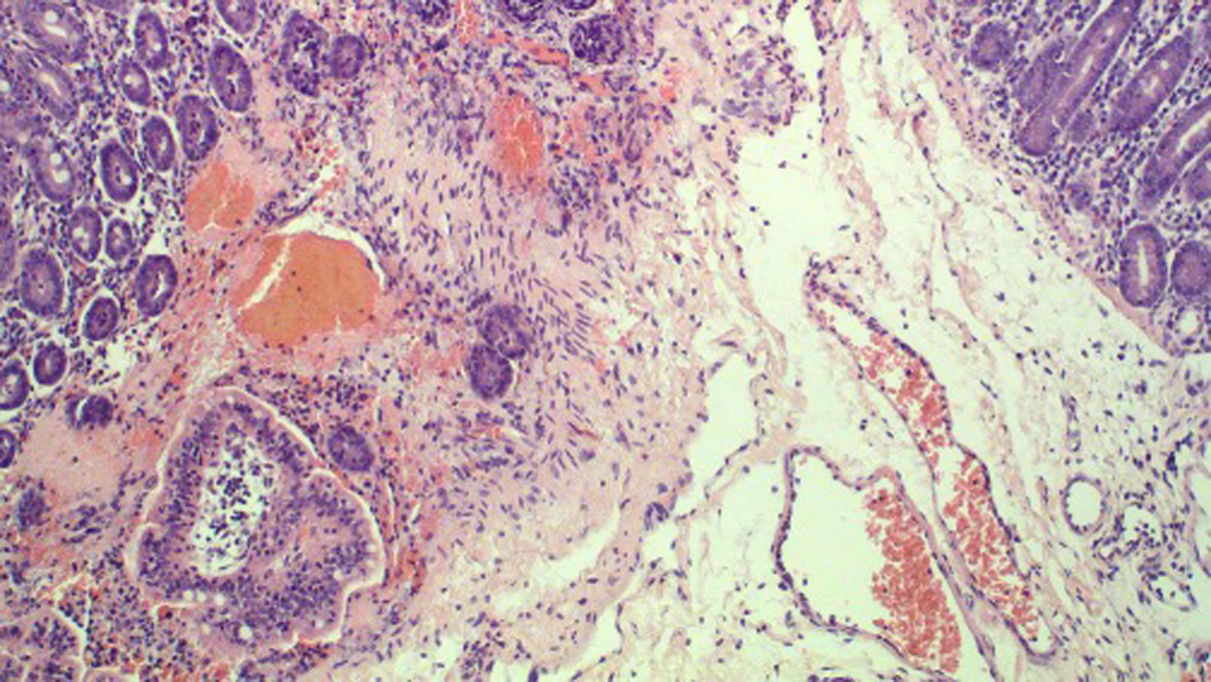
Dilated vessels of the submucosa with the presence of hyaline thrombi.

**Figure 4.**
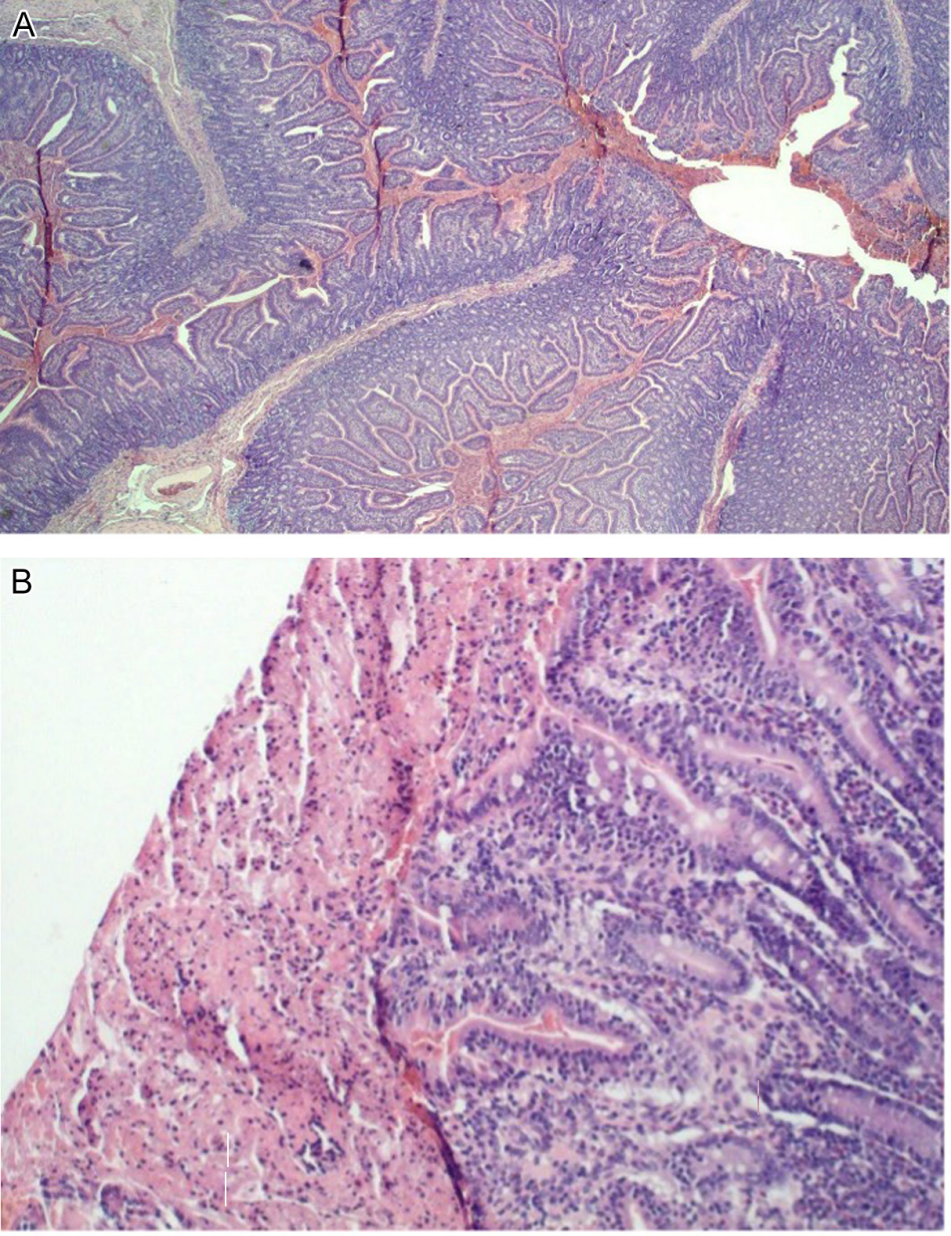
An exudate covering the mucosa was also identified. A (× 25), B (**×** 100).

In total, 1 porcine intestinal segment was resected, and an immediate histopathological examination was performed. Each grasper application point was examined separately (Table 1). Based on the pathological analysis, inflammation of mucosa layer and vascular dilatation of the submucosa layer were present in the small intestine specimens as seen in Figure 2. In Figure 3, dilated vessels of the submucosa with the presence of hyaline thrombi were seen. Microscopically, the bowel segment showed chronic inflammation with a predominance of lymphocytes and plasma cells with few eosinophils, as well as dilatation of the submucosal vascular plexus where small hyaline thrombi were identified in the lumen. The presence of an exudate that was covering the bowel’s mucosa was also observed (Figure 4). The histological findings of all grasper-applied points were similar without significant differences between them. Neither atypical changes nor mucosal necrosis and hyalinization of the lamina propria (features suggestive of ischemic necrosis) was observed.

## Discussion

### Robotic Surgical Systems and Graspers

The advancements in the technology of robotics and minimally invasive surgery have changed dramatically the operative approach to confront urological cancer and other diseases.^6^ The steep learning curve and the good postoperative results have made robotic platforms popular among physicians and surgeons.^7^ Although older robotic systems, such as the da Vinci, have been tested over time, new robotic systems such as avatera still need to be tested concerning their performance for the urological standards for surgery to be clarified. The avatera instruments combine both Bowden-string and in-shaft technologies based on the required actual movement. More precisely, the Bowden-string technology is used for grasping, while in-shaft technology is utilized in all other movements. The avatera grasper is designed and tested for a force range between 6 and 20 N. It is also noteworthy that the actual force depends on the position of the grasper branch. Due to physics laws, the force at the distal tip is normally lower. Moreover, the actual force applied by the graspers depends on the position of the handles of the input devices at the control unit. As for the avatera graspers, there are a lot of benefits that should be discussed upfront. They are single-use; in other words, the patients’ safety is increased 3-fold. First, there is no need for sterilization after surgery and reuse of the graspers from patient to patient. Second, instruments are always new without tear and wear over time, something that secures the best performance level in each operation. Third, if it is necessary there is always an extra grasper to be exchanged within surgery, confronting the very rare cases of material failure. In this study, we aimed to examine and underline the safety of the graspers used by the new avatera robotic surgical system. To our knowledge, this is the first study to evaluate the damage of avatera grasper on bowel segment in a porcine model. An advantage of this study was the in vivo application in a porcine animal model.

### Haptic Feedback

The fact that in the da Vinci surgical systems the grip control mechanism presents an intrinsic resistant force to the surgeon’s fingertips and provides no haptic feedback can be noticed as a negative aspect.^8^ In contrast to open surgery, where the surgeon can feel the resistance and the force applied on tissues, in RALS the surgeon is blind to the real forces applied by the robotic machine. Therefore, the surgeon must know the details of his armamentarium in use and the forces applied by the machine. Unfortunately, such data are rare, given the extent of graspers everyday use.^4^ In addition to this extensive research, a significant effort is ongoing to integrate the instrument–tissue interaction force in minimally invasive surgery, i.e., haptic feedback, which will increase robotic surgery’s safety.^9^

### Technical Details of Graspers and Tissue Damage

In-depth analysis has been realized by Cheng et al^,10^ indicating a correlation between the decrease of tissue damage and the increase of the radius of curvature of the grasper. As expected, the smooth wave pattern reduced tissue damage, unfortunately accompanied by a higher possibility of grasper slipping. The benefit of having a tight grip on the tissue is compensated by the cost of tissue damage and vice versa. This balance depends on the surgeon’s decision-making, bearing in mind the cost and benefit of each grasper. To counteract slipping a larger contact area with a slight profile is favorable. Another study by Heijnsdijk et al^3^ showed that grasper slipping enhances the damaging force. Therefore, graspers with less slipping are safer to use. A recently published study by Huan et al^11^ showed the mechanism with which the softer graspers could reduce tissue damage during RMIS.

### Grasper Handling and Tissue Damage

Grasper handling i.e., the pull force or the axial rotation, are also factors contributing to increased stresses and, thereby, increased tissue injury. The dissociation of the visual axis from the motor axis constitutes an extra difficulty in grasper handling.^12^ Cartmill et al^13^ showed that higher pressure is developed at the graspers’ tips when they are used to retract and deflect tissues at acute angles. The combination of all these factors increases the potential for iatrogenic injury. Interestingly in a study by De et al,^14^ there was no time-dependent association between grasping duration and tissue damage.

### Graspers Force Measurement

In our study, maximal force was applied to the bowel. De et al^14^ showed that stress on a 2-dimensional plane strain model was above 300 kPa beneath the grasper. In their study, a cutoff of 240 kPa for liver tissue was used. The tissue inflammation observed after the grasper used by De et al^14^ aligns with our findings. It remains to be elucidated how many kPa is applied by the avatera grasper. It is obvious that the analysis of grasper force applied on tissue remains a complex topic to measure and quantify from a physics–mathematical perspective. On the other hand, being concentrated on the clinical end effect, the irreversible ischemia characteristics consist of the closest and most realistic approach to objectify any tissue injury.^15^ Another similar study with more technical analysis but without in vivo histological analysis was performed by Khan et al.^16^ After all, since no irreversible damage or ischemia was described under the conditions of maximal force, we can conclude that the use of the new avatera robotic surgical system grasper can be applied with safety on the bowel segments as there is no more damage to be caused.

### Limitations

Our study was not without limitations. First, a pathological investigation could not be performed in patients for medical and safety reasons. Examination of the pathology samples of the sacrificed pig was performed, as its anatomy resembles that of a human. Christensen et al^17^ studied the differences between human and porcine bowel tissue, concluding higher average strength and stiffness with less compliance of human tissue compared to porcine tissue. Another study by Heijnsdijk et al^18^ concluded that the bowel histology is characterized by similar properties among porcine and human bowel tissue but there is also interindividual variability. Second, the small number of pigs and the missing control could be considered a limitation. Best ethical practices and standards such as “refine, reuse, and reduce” underline the importance of using as few animals as possible. Despite the relatively small sample size, we believe that the safety of the technology was adequately interpreted. In addition, we did not investigate the long-term histopathological alterations of the lesions presented in this study. Further investigations should be conducted to evaluate the long-term effect of the aforementioned lesions.

Assuming that there are not many centers possessing the opportunity to perform such experiments, this study constitutes a unique chance to evaluate the safety and efficacy of this technology.

The avatera robotic surgical system is an important surgical tool in RALS. Concerns about the safety and damage caused by graspers on the bowel were elucidated. This experiment on the porcine bowel showed no permanent damage caused by the graspers, except for acute, reversible inflammation. In conclusion, the avatera grasper could be safely used on bowel segments, independent of the applied pressure.

## Figures and Tables

**Table 1.** Pathological Analysis of Each Grasper Application Point

Graspes	Pathological Report
Robotic atraumatic grasper (avateramedical GmbH)	Exudates in the surface, rare epithelial erosions of the superficial villi, and chronic inflammatory infiltrate in the lamina propria were recognized. In the submucosa, a few dilatated vessels are also recognized, and rare polymorphs are seen infiltrating the muscular wall.
Laparoscopic right angle grasper (Richard Wolf GmbH)	Exudates in the surface, rare epithelial erosions of the superficial villi, and chronic inflammatory infiltrate in the lamina propria were recognized. In the submucosa, a few dilatated vessels are also recognized, and rare polymorphs are seen infiltrating the muscular wall.
Laparoscopic curved grasper (Richard Wolf GmbH)	Exudates are found in the bowel’s lumen and the epithelial portion of the mucosa. A mild chronic inflammatory infiltrate is observed in the lamina propria. Rare hyaline thrombi are present in the lumen of small capillaries of the submucosa. Rare polymorphs surround vessels of the muscular wall and small foci of hemorrhage are found in the deep lamina propria.
Laparoscopic atraumatic grasper (Richard Wolf GmbH)	Mild mucosal edema without denudation as well as congestion in the mucosa are recognized. Vascular congestion and microthrombi in the submucosa and rare neutrophils in the muscular wall and subserosa are also detected.
Overall conclusion	Because of the short time of ischemia, no granulation tissue, hemosiderin deposition, or granulomas were found. The bowel specimen showed no atypical reactive changes in its epithelial element, and no fissures or severe endothelial damage, nor hyalinization or fibrosis of the lamina propria, was seen.
